# Mannitol Anaphylaxis in the Setting of Septic Emboli-Induced Intracranial Hemorrhage

**DOI:** 10.7759/cureus.27665

**Published:** 2022-08-04

**Authors:** Barbara M Parker, Vikash Priyadarshi

**Affiliations:** 1 Clinical Pediatric Pharmacy/Clinical Pharmacy, AdventHealth Orlando, Orlando, USA; 2 Family Medicine, Sebastian River Medical Center, Sebastian, USA; 3 Family Medicine, Rockledge Regional Medical Center, Rockledge, USA

**Keywords:** spontaneous intracranial hemorrhage, septic emboli, allergy and anaphylaxis, allergic reaction, bacterial endocarditis

## Abstract

Neurological complications are a significant problem in bacterial endocarditis. Cerebral embolism is the most frequent concern. Acute embolic disease may trigger focal seizures or mycotic aneurysms. Miliary infection is also common, and lumbar puncture can guide in determining the infective organism. Purulent cerebrospinal fluid (CSF) consists often of *Staphylococcus aureus*, a virulent organism, whereas non-virulent organisms (i.e., viridans streptococci) have normal CSF formulae. Microscopic abscesses suggest the potential for aneurysm from bacterial endocarditis amplifying the risk of intracranial hemorrhage. Mannitol and hypertonic (3%) saline are intravenous medications used as a rescue treatment for brain hemorrhage. A patient diagnosed with mycoplasma pneumonia and septic shock secondary to tricuspid endocarditis with extensive pulmonary emboli and metastatic infection to his spine was initiated on antibiotics. He developed a massive intracranial bleed from the rupture of mycotic septic emboli and was given mannitol to decrease intracranial pressure, which caused anaphylaxis.

## Introduction

Infective endocarditis results in cerebrovascular complications in 25%-70% of patients [1]. Septic emboli lead to multiple infarctions of the brain [2]. The cornerstone of treatment is the early initiation of antibiotic therapy. The use of thrombolytics and anticoagulation is avoided in these patients; however, the use of antiplatelet may be considered if indications exist. Intracranial hemorrhage is a typical complication of infective endocarditis as suggested by the presence of two or more cerebral microbleeds on gradient echo sequences. We present a unique case of a patient who developed intracranial hemorrhage from septic emboli due to bacterial endocarditis and suffered an adverse drug reaction (anaphylactic allergy) to mannitol used to treat the brain hemorrhage.

## Case presentation

**Figure 1:**
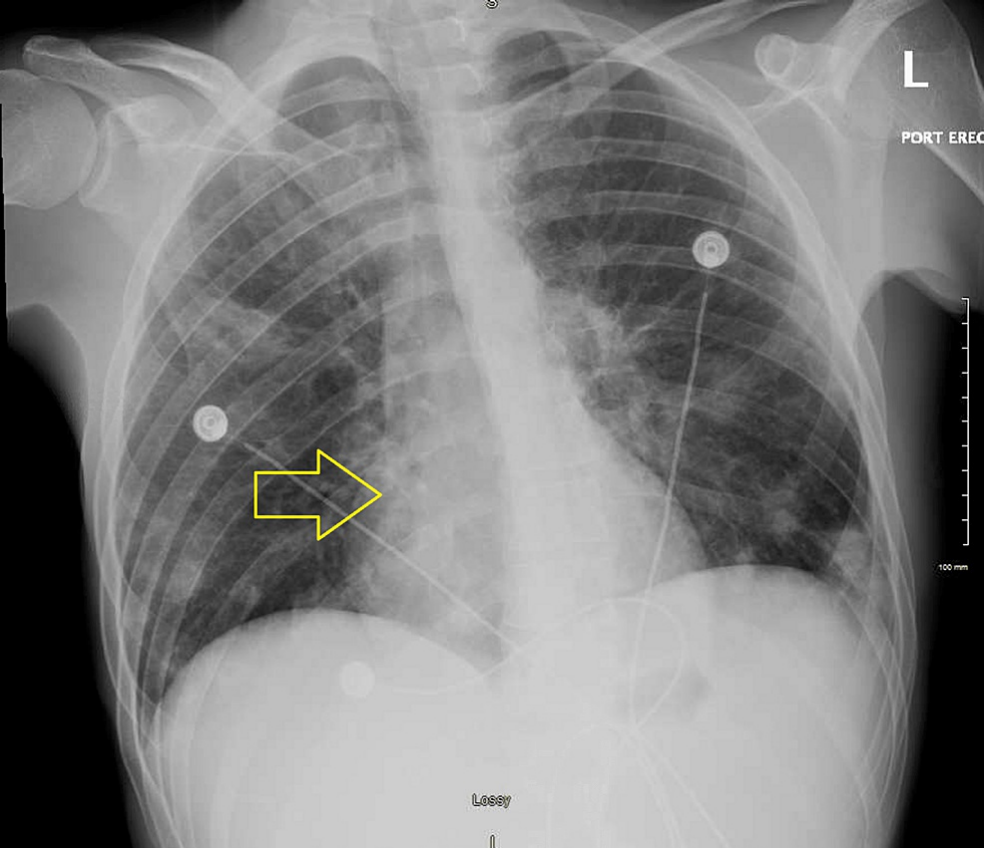
Chest X-ray showing diffuse scattered infiltrates suggesting multilobar pneumonia (arrow)

**Figure 2:**
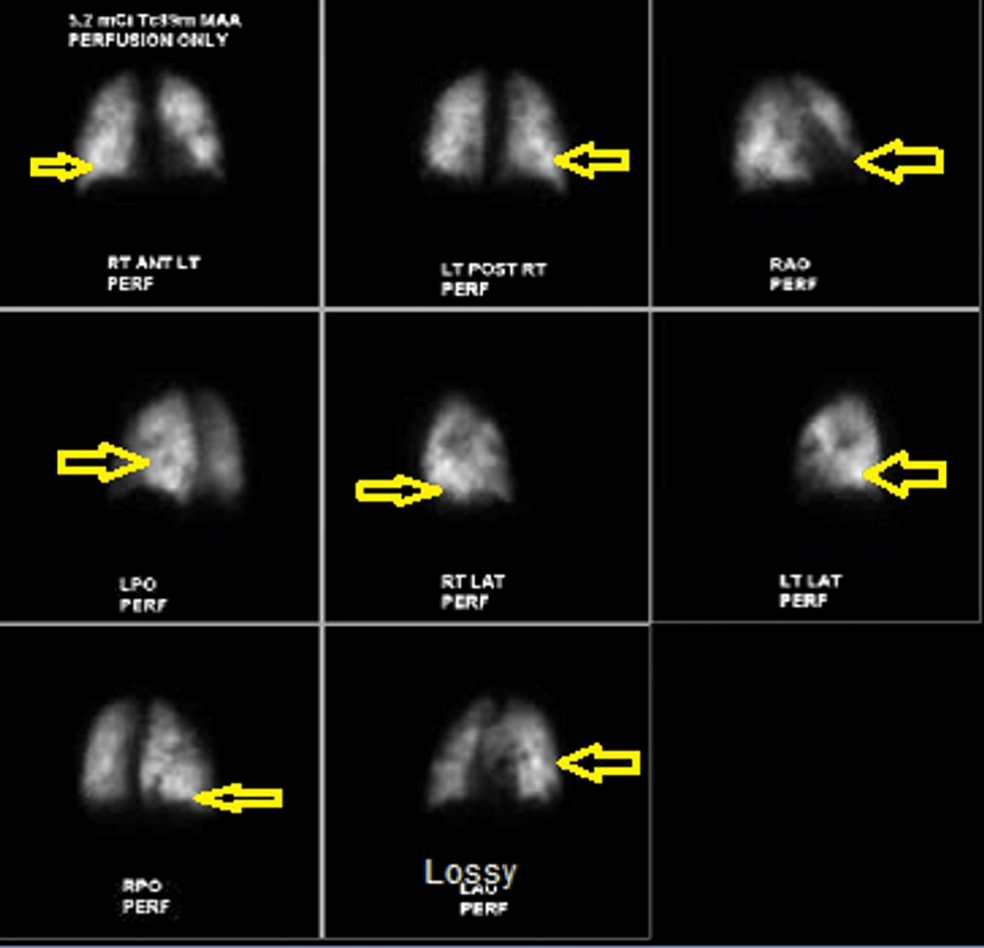
Ventilation/perfusion (VQ) scan showing perfusion defects (arrows)

**Figure 3:**
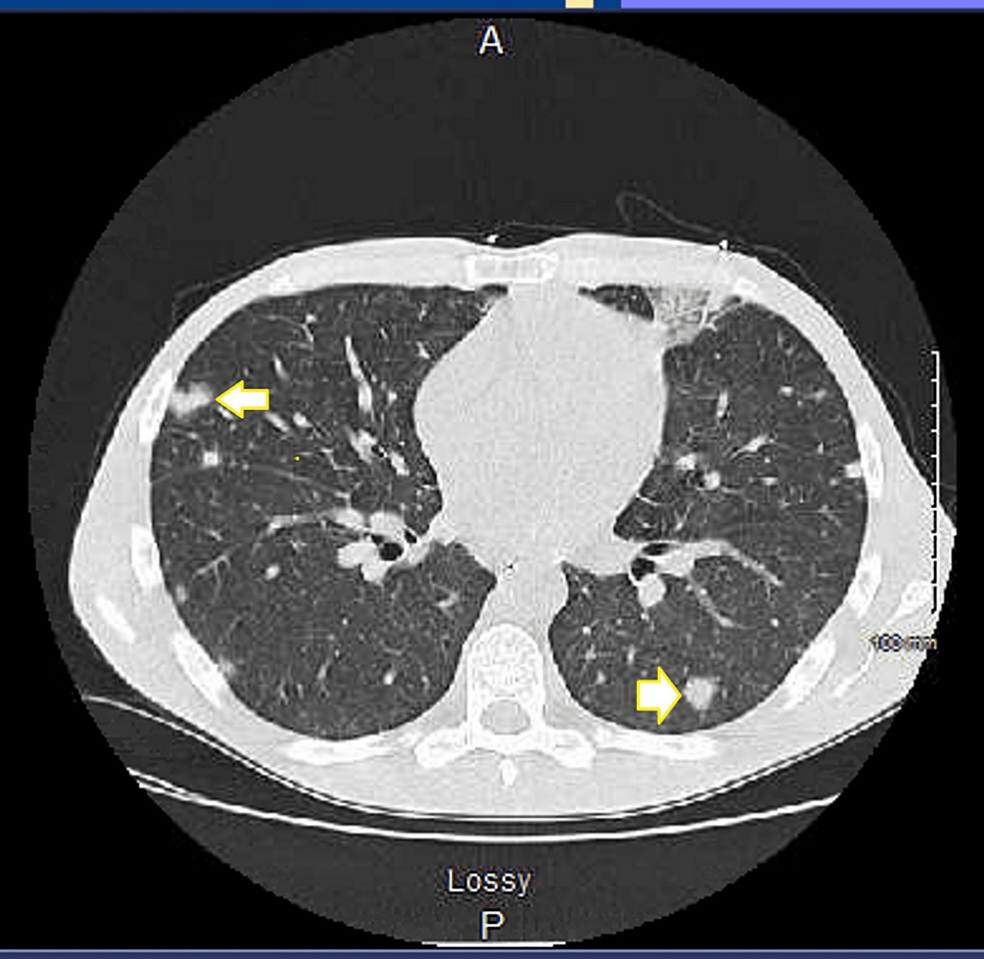
Computed tomography (CT) of the chest with widespread cavitary nodular parenchymal densities consistent with septic emboli (arrows)

**Figure 4:**
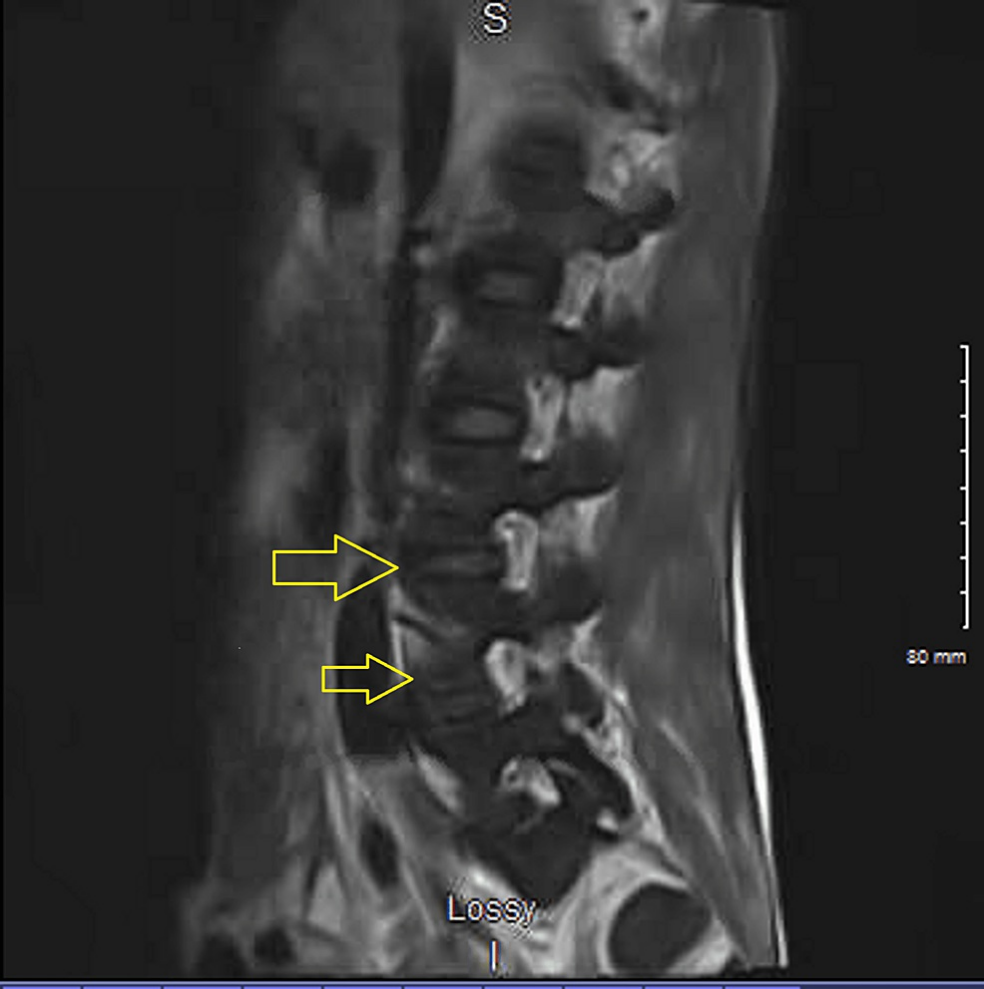
MRI of the spine with lumbar involvement of septic emboli (arrows)

A 26-year-old Caucasian male presented to the emergency department with generalized weakness and progressive lower extremity swelling for two weeks. He also complained of bilateral shoulder pain, dull and achy intermittent chest discomfort with no aggravating factors or radiation of pain, shortness of breath, and lower back pain, none of which he recollects ever having in the past. His past medical history included intravenous drug abuse, hepatitis C, and nicotine dependence. His urine drug screen was positive for opiates, benzodiazepines, and cocaine. A sepsis workup was done due to his initial tachycardia. The patient reported subjective fevers at home. Bilateral lower extremity venous Doppler did not show any deep vein thrombosis (DVT). His vital signs consisted of heart rate in the 110s, blood pressure of 103/64 mmHg with systolic dipping into the low 90s, and breathing on room air. Significant laboratory results included leukocytosis with a white blood cell count of 16,600/μL, neutrophil count of 91.7, acute renal failure with a serum creatinine of 1.9 mg/dL, troponin enzyme of 2.79 ng/mL, elevated C-reactive protein of 577 mg/L (normal reference: 0-9 mg/L), chest X-ray showing diffuse scattered infiltrates (Figure 1), lactic acid within normal limits, platelet of 72,000/μL, hemoglobin of 12.4 g/dL, procalcitonin of 26.96 ng/mL (normal reference: 0-0.08 ng/mL), and elevated alkaline phosphatase of 387 IU/L. His sodium was 133 mg/dL and potassium 5.3 mEq/L. His HIV and hepatitis C panel came back negative. Blood cultures were obtained. The patient was given vancomycin and piperacillin/tazobactam. The initiation of heparin was held due to thrombocytopenia and no risk factors for coronary artery disease. His ventilation/perfusion (VQ) scan showed numerous small perfusion defects (Figure 2). The care team suspected multilobar pneumonia with septic emboli and septicemia, and the patient was transferred to the intensive care unit (ICU). Upon arrival at the ICU, the patient’s antibiotics were switched to linezolid, cefepime, and azithromycin. Computed tomography (CT) scan of the chest revealed widespread cavitary nodular parenchymal densities consistent with septic emboli with lumbar disc involvement (Figures 3, 4), in addition to hepatosplenomegaly. His extremities showed evidence of multiple peripheral punctate lesions consistent with systemic emboli. Blood cultures were positive for Gram-positive cocci in cluster, suggesting *Staphylococcus aureus* infection. The patient was switched to oxacillin for IV drug use methicillin-susceptible *Staphylococcus aureus* (MSSA) tricuspid valve endocarditis and doxycycline for positive *Mycoplasma* IgM for five days. His transthoracic echocardiogram showed tricuspid valve vegetation with an ejection fraction of 65%. A transesophageal echocardiogram was negative for mitral valve regurgitation. The patient was started on methadone to prevent opioid withdrawal.

**Figure 5:**
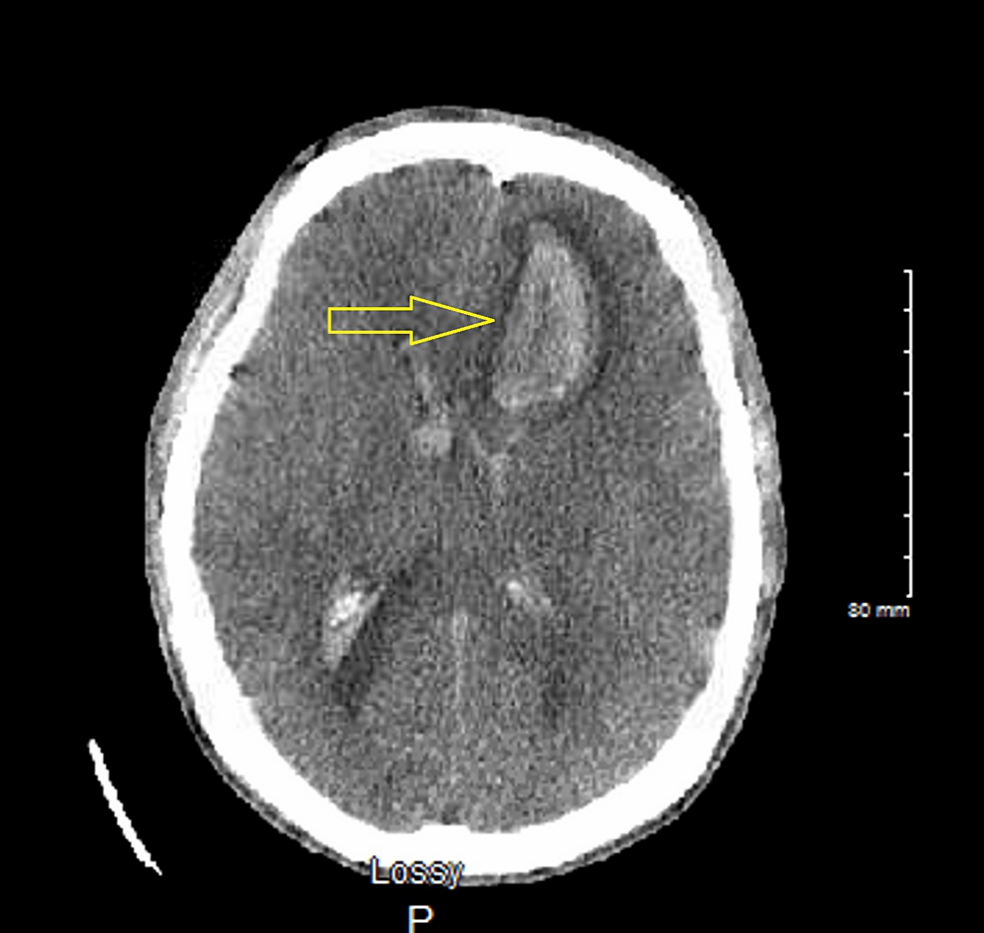
CT of the head showing left frontal intraparenchymal intracranial bleed (arrow)

Four days following admission, the patient developed a spontaneous headache and started seizing from septic emboli to the brain with the involvement of blood vessels causing massive intracranial hemorrhage. CT of the head revealed evidence of a massive left frontal intraparenchymal hemorrhage with associated blood in the ventricles (Figure 5), effacement, and impending herniation. Subarachnoid hemorrhage was in the left more than the right with a midline shift from left to right measuring approximately 9-10 mm with herniation. Cerebral edema was present, and mannitol was administered to decrease intracranial pressure. The patient subsequently had a severe anaphylactic reaction requiring intubation and sedation. He was given methylprednisolone and intravenous diphenhydramine. He became hypotensive and tachycardic and was started on labetalol and nitroprusside drips. He was also given hypertonic (3%) saline at a rate of 50 mL/hour for cerebral edema. Transfer to a higher level of care institution for neurosurgical evaluation was initiated.

## Discussion

Cerebral edema in neurocritical patients is generally treated with hyperosmolar agents (mannitol/hypertonic saline) and corticosteroids [2,3]. Mannitol and hypertonic saline reduce intracranial pressure by producing an osmolar gradient across the blood-brain barrier. A fluid shift follows from the intracellular to the interstitial space into the intravascular space, thus decreasing the volume of fluid within the brain [4]. Hypertonic saline is as effective as mannitol in the treatment of intracerebral hemorrhage [2,5]. Differences between mannitol and hypertonic saline do however exist [4]. Mannitol is a potent diuretic, and repeat doses can cause hypovolemia and hypotension and alter blood rheology [4]. Hypertonic saline has a minimal diuretic effect but can elevate blood pressure [4]. Both mannitol and hypertonic saline (3%) show an immediate reduction in intracranial pressure; however, hypertonic saline may exhibit a slightly longer benefit with a duration of action after infusion termination of up to two hours [5]. Typically, mannitol use concerns are concentration-related, which could cause acute kidney injury [2].

Few cases currently exist in the literature of instances of anaphylactic reaction to mannitol [6]. One case report described a 40-year-old male preparing to undergo retromaxillary tumor resection who, 90 minutes after intubation, received a 100 mL dose of 20% mannitol and minutes later became hypotensive and tachycardic and developed ventricular fibrillation [6]. The patient was resuscitated using CPR, catecholamines, and defibrillation [6]. The IgE-mediated hypersensitivity reaction was due to the release of histamine with its severity linked to mannitol potency [6,7]. Another case occurred in a 39-year-old male following an infusion of 20% mannitol 80 minutes after the beginning of anesthesia [8]. Within 10 minutes, the patient had a systolic blood pressure drop to 40 mmHg, followed by tachycardia and ventricular fibrillation. Immediate resuscitation measures were initiated, and the patient recovered a few hours later without any further events [8]. Intradermal skin testing and in vitro leukocyte histamine release showed a positive specific reaction to 1:100 20% diluted mannitol and 2% mannitol [8]. A third case involved a 60-year-old female with progressive neurological deficits [9]. A computed tomography (CT) scan revealed a right parietal lesion of her brain, which was removed. To relieve intracranial pressure, 150 mL of 20% mannitol was administered [9]. The patient experienced tightness in her chest during mannitol administration. Five days later, her rising intracranial pressure required another administration of 100 mL of mannitol [9]. After the solution was infused, the patient experienced mild respiratory distress, cyanosed lips, and hives on her abdomen [9]. Diphenhydramine and aminophylline were given as supportive treatment and ended her distress [9]. Lastly, a 19-month-old male was given his first cycle of chemotherapy treatment after being given hydration and 20% mannitol to augment hydration and diuresis with cisplatin infusion. After seven minutes of mannitol infusion, the patient developed tachycardia, facial flushing, shortness of breath, cough, rash, and altered mental status. After the infusion was stopped, there was an immediate clinical improvement. The patient was given hydrocortisone, hydroxyzine, and ranitidine without further incident [10].

Studies done in the 1960s suggest that hyperosmolar stimulation can lead to mannitol sensitivity in patients as opposed to an allergic response [10]. If leukocytes are placed under the right in vitro conditions, basophils can release histamine independent of IgE [10]. On the other hand, in patients with known anaphylactoid reactions to mannitol, reactions occur through an IgE-mediated process to stimulate histamine release [10].

## Conclusions

Anaphylactic reactions to mannitol are not common. Hypertonic saline and mannitol are effective agents in the reduction of increased intracranial pressure with brain bleeds. However, more studies are needed to determine the optimal and safest dose concentration, volume, and infusion rate of these agents. Clinicians should remain mindful of the benefits versus risks in the use of these agents and gain a thorough past medical history of patients’ previous exposure and tolerance.

## References

[1] Silver B, Behrouz R, Silliman S (2016). Bacterial endocarditis and cerebrovascular disease. Curr Neurol Neurosci Rep.

[2] Cook AM, Morgan Jones G, Hawryluk GW (2020). Guidelines for the acute treatment of cerebral edema in neurocritical care patients. Neurocrit Care.

[3] Poole D, Citerio G, Helbok R (2020). Evidence for mannitol as an effective agent against intracranial hypertension: an individual patient data meta-analysis. Neurocrit Care.

[4] Pasarikovski CR, Alotaibi NM, Al-Mufti F, Macdonald RL (2017). Hypertonic saline for increased intracranial pressure after aneurysmal subarachnoid hemorrhage: a systematic review. World Neurosurg.

[5] Qureshi AI, Wilson DA, Traystman RJ (1999). Treatment of elevated intracranial pressure in experimental intracerebral hemorrhage: comparison between mannitol and hypertonic saline. Neurosurgery.

[6] Schmid P, Wüthrich B (1992). Peranaesthetic anaphylactoid shock due to mannitol. Allergy.

[7] Hegde VL, Venkatesh YP (2004). Anaphylaxis to excipient mannitol: evidence for an immunoglobulin E-mediated mechanism. Clin Exp Allergy.

[8] Biro P, Schmid P, Wüthrich B (1992). [A life-threatening anaphylactic reaction following mannitol]. Anaesthesist.

[9] McNeill IY (1985). Hypersensitivity reaction to mannitol. Drug Intell Clin Pharm.

[10] Lightner DD, De Braganca K, Gilheeney SW, Khakoo Y, Kramer K, Balas M (2013). A case of mannitol hypersensitivity. J Pediatr Hematol Oncol.

